# Does a surgical helmet provide protection against aerosol transmitted disease?

**DOI:** 10.1080/17453674.2020.1771525

**Published:** 2020-06-23

**Authors:** Max Joachim Temmesfeld, Rune Bruhn Jakobsen, Peter Grant

**Affiliations:** a Department of Orthopedic Surgery, Akershus University Hospital, Lørenskog, Norway;; b Department of Health Management and Health Economics, Institute of Health and Society, University of Oslo, Norway;; c Department of Orthopaedics, Institute of Clinical Sciences Sahlgrenska Academy, University of Gothenburg, Sweden;; d Lovisenberg Diaconal Hospital, Oslo, Norway

## Abstract

Background and purpose — The COVID-19 pandemic caused by infection with SARS-CoV-2 has led to a global shortage of personal protective equipment (PPE). Various alternatives to ordinary PPE have been suggested to reduce transmission, which is primarily through droplets and aerosols. For many years orthopedic surgeons have been using surgical helmets as personal protection against blood-borne pathogens during arthroplasty surgery. We have investigated the possibility of using the Stryker Flyte surgical helmet as a respiratory protective device against airborne- and droplet-transmitted disease, since the helmet shares many features with powered air-purifying respirators.

Materials and methods — Using an aerosol particle generator, we determined the filtration capacity of the Stryker Flyte helmet by placing particle counters measuring the concentrations of 0.3, 0.5, and 5 µm particles inside and outside of the helmet.

Results — We found that the helmet has insufficient capacity for filtrating aerosol particles, and, for 0.3 µm sized particles, we even recorded an accumulation of particles inside the helmet.

Interpretation — We conclude that the Stryker Flyte surgical helmet should not be used as a respiratory protective device when there is a risk for exposure to aerosol containing SARS-CoV-2, the virus causing COVID-19, in accordance with the recommendation from the manufacturer

The rapid development of the COVID-19 pandemic has led to severe shortages around the globe of personal protective equipment (PPE) for healthcare personnel such as regular surgical masks, tight-fitting masks (filtering facepieces [FFP]), protective eyeglasses, and face shields (Kamerow 2020, World Health Organization 2020). The virus causing COVID-19, SARS-CoV-2, is believed to spread primarily through droplets and aerosol in the immediate vicinity of an infected person (Bahl et al. 2020). A recent study showed that SARS-CoV-2 aerosols remain airborne and viable for at least 3 hours in closed spaces, thus raising the concern of airborne transmission (van Doremalen et al. 2020). A recent review also discussed the transmission of viral particles from aerosolized body fluids by using power drills, pulsed lavage, and other equipment during surgery (Basso et al. 2020). This has not been reported for SARS-CoV-2, but it is conceivable and has been shown in vitro for other viruses (Johnson and Robinson 1991, Garden et al. 2002).

Numerous alternative concepts of respiratory PPE have been suggested, for example the use of powered air-purifying respirators (PAPR). In these devices, filtered air is drawn by an electric fan into a closed helmet. Even though PAPRs offer superior protection compared with standard FFPs, hospitals would have to pay a lot to commercially acquire a sufficient number of PAPRs to equip their healthcare personnel. Additionally, a shortage of PAPRs is to be expected during a pandemic.

Surgical helmets with internal electric fans share many features of a PAPR and were suggested as an alternative during the SARS epidemic in China in 2003 (Ahmed et al. 2005). Such helmets are regularly used in orthopedic arthroplasty surgery. The hood of the surgical helmet is air-permeable over the fan intake, while the rest of the hood material is practically air impermeable. The original purpose of surgical helmets was to protect patients from particles that the surgical team might emit into the wound. Additionally, surgical helmets will protect the surgeon from direct body fluid contamination.

The Flyte model (Stryker Instruments, Kalamazoo, MI, USA) is commonly used in Scandinavia, continental Europe, and the United States and thus available in many Western hospitals. We investigated the protective abilities of this helmet in the context of the ongoing COVID-19 pandemic as part of an ongoing research project, which aims to convert surgical helmets into PAPRs with the help of specialized filters. 

## Materials and methods

**Figure 1.**
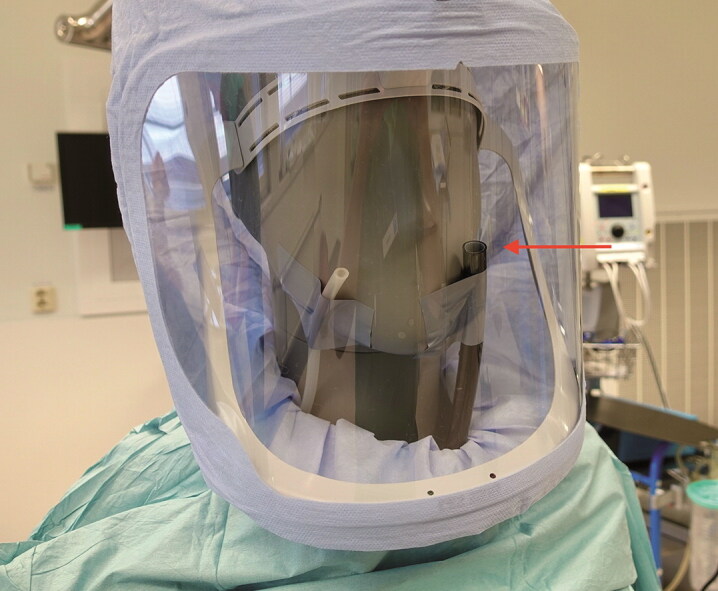
Test setup. The dummy with helmet, hood, and gown in the test setup. Arrow indicating particle counter inside helmet. (White probe is a passive pressure probe not used for tests reported in this paper.)

**Figure 2.**
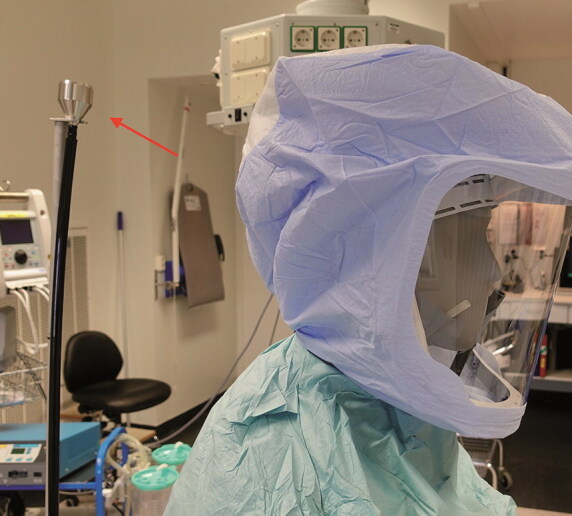
Test setup. Arrow indicating particle counter outside helmet.

The investigation was performed in an orthopedic operation theatre at Sahlgrenska University Hospital, Gothenburg, Sweden. The theatre is equipped with a mixed ventilation system that meets SIS-TS 39:2015 requirements and is audited annually (Swedish Standards Institute 2015). A Stryker Flyte helmet (Stryker Instruments, Kalamazoo, MI, USA) with the standard hood (Flyte Hood, product no. 0408-800-000) was mounted onto a dummy with head and torso. A standard surgical gown (Barrier Surgical Gown Classic, Mölnlycke, Sweden) was tightened around the neck. A 6D Laskin nozzle aerosol generator (Air Techniques International, Owings Mills, MD, USA) generated an oil-based hydrogenated 1-Decene homopolymer (PAO-4) test aerosol. The generator was active for approximately 15 seconds at the start of each test. A particle counter (Solair 3100, Lighthouse, Fremont, CA, USA) detector probe was fastened to the nose of the dummy (Figure 1). Another identical particle counter was positioned approximately 20 cm adjacent to the fan intake outside the hood (Figure 2). The concentrations of 0.3, 0.5, and 5µm particles per cubic foot inside and outside the surgical helmet were continuously recorded with synchronized dataloggers.

At first, the helmet fan was set to maximum speed, and the dummy was left for 30 minutes to establish a steady-state of ambient particles and to minimize the risk of false readings due to particles released from the hood and dummy.

We performed tests with the fan at the minimum and the maximum speed. Each test lasted between 15 and 30 minutes. The total inward leakage (TIL) was calculated with the following formula:

TIL =(Particle concentration inside helmet)/(Particle concentration outside helmet (ambient))

Reciprocally, the filtration efficiency (FE) was calculated as follows:

FE = 1–TIL

Particle concentrations were converted to metric units and the data were summarized and visualized with descriptive statistics (area under the curve [AUC], mean, and 95% confidence intervals [CI]) using Prism 8 (Graphpad Software, San Diego, CA, USA).

### Funding and potential conflicts of interest

This work was performed as an ongoing project to research whether surgical helmets can be modified safely for use during the ongoing pandemic, with a patent pending for a retrofitted adapter. The project did not receive any specific funding.  

## Results

**Figure 3.**
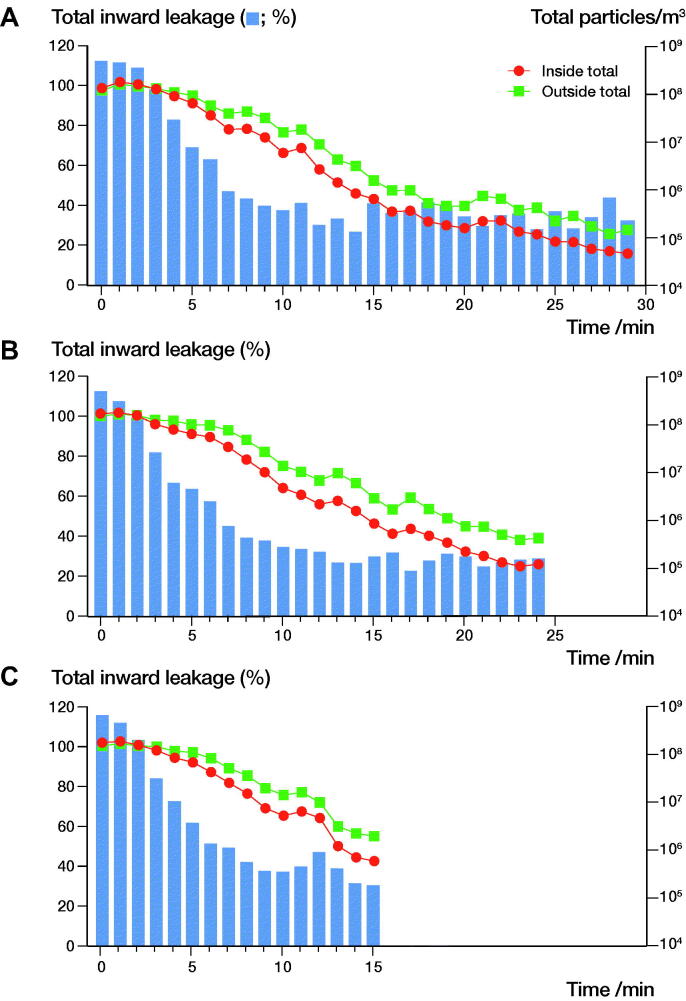
Line plots showing the total particles per m^3^ for the outside counter (green) and inside counter (red) versus time in minutes with bars showing the calculated percentage total inward leakage (TIL) at every time point. Each panel represents an individual experiment. Top panel with fan at maximum speed and the other 2 with fan at minimum speed.

The filtration efficiency (FE) for the total number of particles of all measured sizes was 19% (CI 7.2–31), corresponding to a total inward leakage (TIL) of 81%. The FE was statistically significantly lower for smaller sized particles and for the smallest particles of 0.3 µm the FE was particularly poor at 3.3% (CI –7.6 to 14) (Table 1). At declining ambient particle concentrations outside the helmet, the TIL also decreased in all experiments (Figure 3).

**Table:** Particle counts inside and outside and filtration efficiency of the Stryker Flyte helmet

		Particle size		
	0.3 µm	0.5 µm	5 µm	Total
	n × 10^7^	n × 10^7^	n	n × 10^7^
Inside helmet	1.67	0.60	439	2.27
(95% CI)	(1.60–1.75)	(0.52–0.68)	(214–664)	(2.12–2.25)
Outside helmet	1.73	1.08	2,823	2.81
(95% CI)	(1.58–1.89)	(0.93–1.24)	(1,180–4,467)	(2.51–3.12)
FE (%)	3.3	44	84	19
(95% CI)	(–7.6 to 14)	(31–57)	(80–89)	(7.2–31)

CI, confidence interval, FE, filtration efficiency

**Figure 4.**
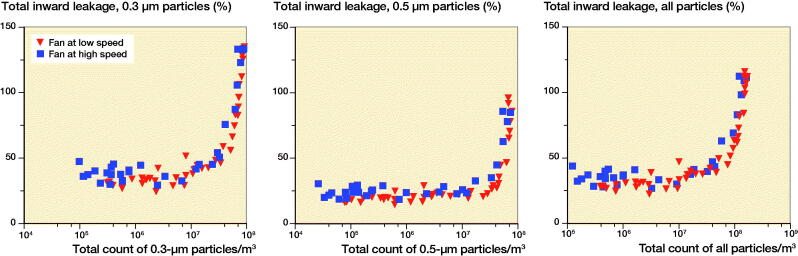
Scatterplots of percentage total inward leakage versus total count of 0.3 µm, 0.5 µm, and all particle sizes per m^3^.

Nevertheless, the TIL for the 0.3 µm size aerosols regularly exceeded 100%, indicating an accumulation of particles inside the helmet when the outside particle concentration exceeded approximately 7 × 10^6^/m^3^. This was not the case for the 0.5 µm size aerosols, yet the TIL did approach 100% at the highest concentrations of particles outside the helmet (Figure 4). The filtration efficiency for the largest particles (5.0 µm) was markedly higher than for smaller particles (Table). The absolute numbers of large particles were few compared with the smaller sizes and did not markedly influence the TIL for the total number of particles (Figure 4).  

## Discussion

This study supports the recommendation by the manufacturer not to use the regular Stryker Flyte helmet as respiratory protection equipment against SARS-CoV-2 (Stryker Corporation, 2020). The filtration efficiency for particles of all measured sizes is low (19%). Our data indicate an accumulation of 0.3 µm particles inside the helmet, when ambient concentrations are high. In the light of this, wearing the Stryker Flyte helmet as the only respiratory PPE is not advisable.

Comparing the TIL for 0.3 µm and 0.5 µm (97% and 56%) in the present study with regular respiratory protective equipment such as FFPs further emphasizes that the Flyte helmet is by no means effective as protective equipment against aerosol-transmitted disease. Maximum permitted TIL for FFPs ranges from maximum 22% for an FFP-1 to 2% for FFP-3 (EU-standard EN149:2001), the latter being the recommended FFP to use for COVID-19 patients during aerosol-generating procedures. However, also FFPs have inherent problems and depend on a tight fit to the face of the user to reduce air bypassing the filter. Maximum protection is achieved using a PAPR equipped with an approved filter. It is imaginable that regular surgical helmets could be modified with proper filters to achieve sufficient FE. The protective ability of surgical hoods to safeguard the surgeon against exposure to infectious bodily fluids and direct transfer of microorganisms or particulate matter has been verified in vitro (Wendlandt et al. 2016). The hood on the Flyte Personal Protection System provides leading-class AAMI/ANSI Level 4 protection (Association for Advancement of Medical Instrumentation/American National Standards Institute); nevertheless, the top of the hood that the air passes through is not designed to filter aerosols. Our findings are in line with a previous study from 2004 that evaluated the respiratory protective properties of 2 types of surgical helmets compared with an N100 filtering facepiece respirator combined with a surgical mask and full face shield (Derrick and Gomersall 2004). In that study they found ratios of ambient particle concentration to particle concentration inside the helmet to fall between 2 and 5, which corresponds to a TIL of 20–50%.

There are several limitations to this investigation. First, the experimental setup in this study did not comply with any formal regulatory standards. Second, we tested artificially produced aerosol particles of 3 predefined sizes and not virus-containing particles as is sometimes performed (Fabian et al. 2008, Makison Booth et al. 2013). Third, it is likely that particles from a sneeze may be substantially larger than the particles tested in our setup (Han et al. 2013). However, the setup we constructed was deliberately similar to real-life situations with COVID-19 patients in the operation theatre. We used a PAO-4 test aerosol, which is FDA approved for regulated filter leakage testing. The particles we tested were of the same size as found in aerosols of healthy patients and patients with influenza during coughing, assisted and regular breathing where a majority of particles have been found to be less than 1 µm (Papineni and Rosenthal 1997, Yang et al. 2007, Fabian et al. 2008, Wan et al. 2014). Most testing and certification protocols for respiratory protective equipment use very high concentrations of particles in the range of 7–10 × 10^11^/m^3^ (Derrick and Gomersall 2004, Gawn et al. 2008, Makison Booth et al. 2013). Even the highest concentrations of particles generated in our study were markedly lower than in other published studies. We consider it a strength that we used a maximal concentration of particles several orders of magnitude lower (∼1,6 × 10^9^/m^3^) and could still demonstrate substantial inward leakage.

We conclude that the Stryker Flyte surgical helmet does not provide sufficient protection against aerosol transmitted diseases. It is important to comply with the instructions of the manufacturer that the Stryker Flyte surgical helmet should not be used as a respiratory protective device against COVID-19.
